# Comparison of developing tuberculosis following tumor necrosis factor inhibition and interleukin-6 inhibition in patients with rheumatoid arthritis: a nationwide observational study in South Korea, 2013–2018

**DOI:** 10.1186/s13075-022-02842-6

**Published:** 2022-06-27

**Authors:** Seung Min Jung, Minkyung Han, Eun Hwa Kim, Inkyung Jung, Yong-Beom Park

**Affiliations:** 1 Division of Rheumatology, Department of Internal Medicine, School of Medicine, The Catholic University of Korea, Seoul, Republic of Korea; 2 Department of Biomedical Systems Informatics, Biostatistics Collaboration Unit, Yonsei University College of Medicine, Seoul, Republic of Korea; 3 Division of Biostatistics, Department of Biomedical Systems Informatics, Yonsei University College of Medicine, 50-1 Yonsei-ro, Seodaemun-gu, Seoul, 03722 Republic of Korea; 4 Division of Rheumatology, Department of Internal Medicine, Institute for Immunology and Immunologic Disease, Yonsei University College of Medicine, 50-1 Yonsei-ro, Seodaemun-gu, Seoul, 03722 Republic of Korea

**Keywords:** Biologic therapy, Rheumatoid arthritis, Tuberculosis, TNF inhibitors, Tocilizumab, Latent tuberculosis

## Abstract

**Background:**

Tumor necrosis factor (TNF) inhibitors increase the risk of tuberculosis (TB) in patients with rheumatoid arthritis (RA). This study compared the incidence of TB after treatment with TNF inhibitors and tocilizumab in patients with RA, separately in those who were treated for latent tuberculosis infection (LTBI) and those without evidence of LTBI.

**Methods:**

This study included patients with RA who initiated TNF inhibitors and tocilizumab between December 2013 and August 2018. Patient data were collected from the nationwide database of the Health Insurance Review and Assessment service in South Korea. The incidence of TB was compared among different biologic drugs in patients with or without LTBI treatment.

**Results:**

Of 4736 patients, 1168 were treated for LTBI and 48 developed TB (554.9 per 100,000 person-years). When compared based on etanercept, infliximab showed a higher risk of TB (adjusted incidence rate ratio 2.71, 95% confidence interval 1.05–7.01), especially in patients without evidence of LTBI. Other TNF inhibitors and tocilizumab showed a comparable incidence of TB, regardless of treatment for LTBI. There was no significant difference in TB incidence after biologic therapy between patients with and without LTBI treatment (627.9/100,000 vs. 529.5/100,000 person-years). In patients treated for LTBI, no differential risk of TB was observed among biologic drugs.

**Conclusions:**

The incidence of TB was not significantly different among biologic drugs in the current era, except for infliximab in patients who were not treated for LTBI. Treatment of LTBI might alleviate the drug-specific risk of TB in patients with RA.

**Supplementary Information:**

The online version contains supplementary material available at 10.1186/s13075-022-02842-6.

## Introduction

Rheumatoid arthritis (RA) is a chronic inflammatory disease requiring long-term anti-inflammatory therapy. The treatment of RA based on the use of disease-modifying anti-rheumatic drugs (DMARDs) has considerably developed over the last 100 years [1, 2]. Recently, the introduction of biologic therapy targeting inflammatory cells and cytokines has greatly improved the clinical outcome of patients with RA. However, increased risk of infection has been reported, probably due to disease-associated immune dysfunction and sustained immunosuppressive therapy [3–5].

Since the late 1990s, biologic agents that inhibit the action of tumor necrosis factor (TNF)-α have been approved for the treatment of RA. Despite the good therapeutic efficacy of anti-TNF therapy, the increase in infection has been the main concern in terms of safety [6–10]. The association between TNF blockade and tuberculosis (TB) has been established in the early stage of biologic therapy [11, 12]. Patients treated with TNF inhibitors showed a significantly higher incidence of TB than those naïve to biologic agents. Furthermore, observational studies revealed the differential risk of TB development among anti-TNF drugs [13–15]. Monoclonal antibodies, such as infliximab and adalimumab, had a greater effect on TB development than the receptor-Fc fusion protein etanercept. However, in the current era of biologic therapy, several issues remain to be determined. On the basis of the significant link between anti-TNF therapy and TB, the current guidelines strongly recommend performing the appropriate test to detect latent TB infection (LTBI) and applying prophylactic therapy for LTBI before the use of biologic drugs [16, 17]. In this clinical setting, it is unclear whether the differential risk of TB among biologic drugs still exists, particularly in patients treated for LTBI. Moreover, biologic agents have diversified over the last decade [2, 18]. Five TNF inhibitors have been approved for the treatment of RA, and biologic agents with different modes of action have been widely used in patients with RA. However, the comparative risk of TB has not been evaluated in patients treated with the latest anti-TNF agent and non-TNF biologic agents.

South Korea is a country of intermediate TB burden with a high prevalence of LTBI, especially in older generations [19]. The incidence of TB was 77 per 100,000 persons in South Korea, showing the highest among the member countries of the Organization for Economic Cooperation and Development [20]. Although the TB incidence is steadily decreasing, it is much higher than that in Western countries [21]. Recent study using interferon-gamma releasing assay showed that LTBI was noted in 20.3% of the total population with the highest prevalence of 42% in age group ≥ 60 years [19]. Assessment of TB risk would be more valid in countries of intermediate TB burden than in countries with low TB incidence.

In this study, we compared the risk of TB in patients with RA treated with four TNF inhibitors and tocilizumab in South Korea with an intermediate TB burden. The nationwide data were collected from the national health insurance claims database, and the risk of TB after biologic therapy was evaluated separately in patients who were treated for LTBI and patients without evidence of LTBI.

## Patients and methods

### Patient inclusion

This study recruited all domestic patients with RA who were treated with TNF inhibitors or tocilizumab between July 2013 and April 2018 from a national database maintained by the Health Insurance Review and Assessment (HIRA) service. Health insurance in South Korea is a form of social security system provided to all citizens, and all health-care institutions submit each individual’s data on diagnosis and treatment for insurance claims to HIRA. Thus, the HIRA database contains the detailed health-care information of all citizens. In South Korea, health insurance covers biologic therapy in patients with RA if they exhibit moderate to high disease activity despite treatment with two or more conventional DMARDs for > 6 months. During the study period, TNF inhibitors (etanercept, infliximab, adalimumab, and golimumab) and tocilizumab have been available as first-line biologic agents in patients with RA who have inadequate response to two or more conventional DMARDs.

Patients were selected from the HIRA database using the drug code of TNF inhibitors and tocilizumab, along with the code of seropositive RA (M05.8 and M05.9) in the revised 10th International Classification of Diseases. We included only patients who started TNF inhibitors or tocilizumab between July 2013 and April 2018. The study population was further determined based on the following exclusion criteria: (1) use of TNF inhibitors or tocilizumab for a main diagnosis other than RA (e.g., ankylosing spondylitis or psoriatic arthritis), (2) treatment with other biologic agents within 6 months before the use of a biologic drug of interest, and (3) treatment of TB or nontuberculous mycobacterial infection between Jan 2013 and the introduction of TNF inhibitors or tocilizumab.

The patient information in the HIRA database is anonymized to ensure confidentiality. This study was approved by the Institutional Review Board of Yonsei University College of Medicine (4–2018-0983) and reviewed by the HIRA Research Committee.

### Data collection

Demographic data including age, sex, and entry year were collected from the HIRA database. The details on drug use were inspected in all included patients. The main interests were the use of biologic agents and anti-TB drugs. The use of corticosteroids and conventional DMARDs, such as methotrexate, leflunomide, and tacrolimus, was also investigated to evaluate the effect of immunosuppressive medications on TB. Patients were followed up until the occurrence of TB, withdrawal of biologic therapy, or end of the study period.

### Definition of LTBI and TB

The incidence of TB and the treatment of LTBI were defined according to the use of anti-TB drugs. Biologic therapy and anti-TB treatment are under special surveillance in Korean medical system, and thus, these treatments would not be omitted from the national database. We assumed that anti-TB treatment is the most accurate indicator for the diagnosis of active TB. We inspected the types of anti-TB drugs and the temporal relationship between biologic therapy and anti-TB treatment. The anti-TB drugs of interest were isoniazid, rifampin, pyrazinamide, and ethambutol. In South Korea, diagnostic evaluation and treatment for LTBI are mandatory in all patients before biologic therapy. Thus, treatment of LTBI was defined as prescription of isoniazid and/or rifampin before or simultaneously with the use of a biologic drug. Further, treatment of TB was determined by the combination of three or more anti-TB drugs after the initiation of biologic therapy based on the standard regimen. If TB occurred during biologic therapy or within 3 months after stopping biologic therapy, TB was considered to be related to biologic therapy. However, if the time interval between biologic withdrawal and TB occurrence was > 3 months, TB was considered to be irrelevant to biologic therapy. In patients who switched biologic drugs (i.e., switchers), TB within 3 months after the last injection of the previous biologic drug was more likely to be associated with the previous drug. However, it is unclear which drug is more relevant to TB in switchers. Thus, case of TB was separately evaluated in patients excluding switchers and in all patients including switchers (the data of all patients including switchers are presented in the Supplement).

### Statistical analysis

Baseline characteristics were presented as mean ± standard deviation for continuous variables and frequencies (percentages) for categorical variables. The differences among biologic drugs were compared using one-way analysis of variance and the chi-square test. We estimated the incidence of TB in each group as cases per 100,000 person-years and performed a Poisson regression analysis with an offset for person-years to obtain the incidence rate ratio (IRR) and 95% confidence interval (CI). We adjusted the incidence of TB for age, sex, and calendar year. Due to the low incidence of TB, no further adjustments for the use of glucocorticoid and DMARD were not presented in this study. The cumulative incidence of TB after biologic therapy was calculated using the Kaplan–Meier method, and the differences among groups were determined using the log-rank test with multiple comparison adjustment. We further assessed the IRRs stratified by LTBI treatment and follow-up duration (< 0.5, 0.5–1, 1–3, and ≥ 3 years). All analyses were performed using the SAS Enterprise Guide (SAS Institute Inc., Cary, NC). The results were considered as statistically significant at *P* < 0.05.

## Results

### Patient selection and TB development

**Fig. 1:**
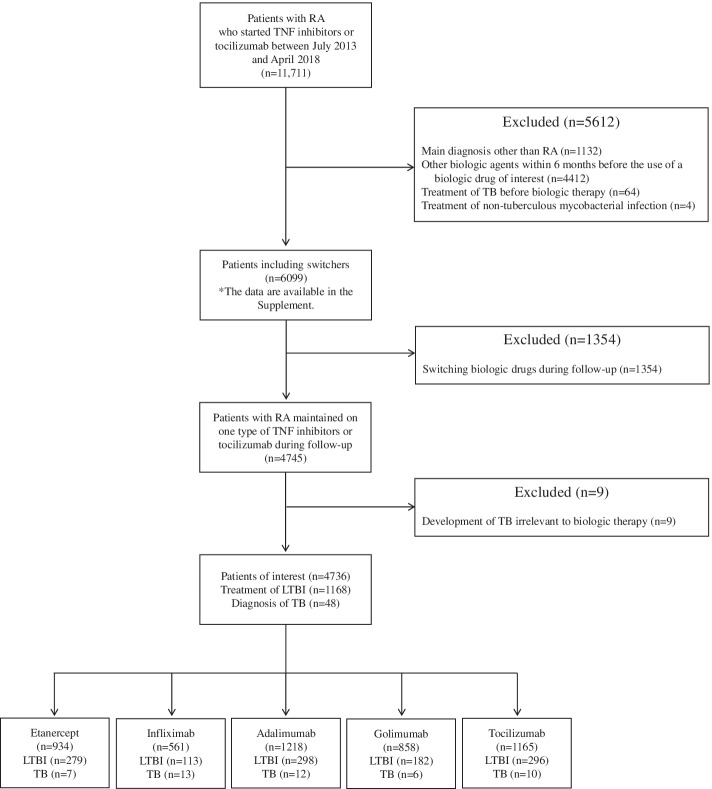
Flowchart of patient selection. Patients with rheumatoid arthritis (RA) who started tumor necrosis factor (TNF) inhibitors or tocilizumab between December 2013 and April 2018 were selected from a nationwide database maintained by the Health Insurance Review and Assessment service. According to the exclusion criteria, 6099 patients were selected for the analysis (the incidence of tuberculosis [TB] in these patients including switchers are presented in the Supplement). To avoid the unclear association between biologic agents and TB in patients who switched biologic drugs, we analyzed the incidence of TB in patients who were treated with only one type of TNF inhibitor or tocilizumab during the follow-up. Further, patients who developed TB at > 3 months after stopping the biologic drug were excluded from the analysis because TB was considered to be irrelevant to biologic therapy in these patients. A total of 4736 patients with RA were included for the final analysis RA, rheumatoid arthritis; TNF, tumor necrosis factor; TB, tuberculosis; LTBI, latent tuberculosis infection

The patient inclusion process is presented in Fig. 1. We selected 11,711 patients who started TNF inhibitors or tocilizumab during the study period under the diagnostic code of RA. We inspected the main diagnosis indicated for biologic therapy and the medications before starting and after stopping biologic therapy. Patients who started biologic therapy for the treatment of an inflammatory disease other than RA were excluded from the analysis (*n* = 1132). Patients with a history of other biologic therapy within 6 months before starting biologic therapy (*n* = 4412) and those who switched biologic drugs during the study period (*n* = 1354) were also excluded. There were 64 and 4 patients treated for TB and nontuberculous mycobacterial infection, respectively, from Jan 2013 to the introduction of biologic therapy. New case of TB was found in 57 patients who started TNF inhibitors or tocilizumab during the study period. Among the 57 patients, 9 cases of TB were observed at > 3 months after the last injection of biologic drugs, and TB was considered to be irrelevant to biologic therapy in these patients. After excluding these 9 patients, 4736 patients were included in the final analysis, and 48 cases of TB were observed.

### Patient characteristics

The patient characteristics are presented in Table 1. During the study period, the most commonly initiated biologic agent was adalimumab (25.7%), followed by tocilizumab (24.5%), etanercept (19.7%), golimumab (18.1%), and infliximab (11.8%). The mean age was 54.2 years, and 81.1% of the patients were women. There were differences in age and sex depending on the biologic agent used. We investigated the concomitant use of conventional DMARDs or glucocorticoids during the initial 3 months of biologic therapy. The most commonly used DMARD was methotrexate, which was combined with biologic therapy in 3953 (83.5%) patients. Methotrexate was administered less frequently to patients treated with etanercept and tocilizumab. Use of leflunomide and tacrolimus was found in 678 (14.3%) and 212 (4.5%) patients, respectively. Glucocorticoids were prescribed in 4143 (87.5%) patients and were more frequently used in the tocilizumab group. Treatment of LTBI was more prevalent in the etanercept group (29.9%), whereas the infliximab group included fewer patients who were treated for LTBI (20.1%). Etanercept and infliximab were more likely to be started at the earlier phase of the study period than the other biologic agents.

**Table 1:** Clinical characteristics of patients with rheumatoid arthritis in a nationwide database

		Anti-TNF					
	All (*n* = 4736)	ETA (*n* = 934)	INF (*n* = 561)	ADA (*n* = 1218)	GOL (*n* = 858)	TOC (*n* = 1165)	*P* value
Age (years), mean ± SD	54.2 ± 13.3	53.4 ± 14.3	56.2 ± 12.7	52.3 ± 13.6	55.0 ± 12.8	55.5 ± 12.6	< 0.0001
Women, *n* (%)	3841 (81.1)	747 (80.0)	471 (84.0)	947 (77.8)	708 (82.5)	968 (83.1)	0.0021
Combination of DMARDs, *n* (%)^a^							
Methotrexate	3953 (83.5)	768 (82.2)	485 (86.5)	1047 (86.0)	772 (90.0)	881 (75.6)	< 0.0001
Leflunomide	678 (14.3)	89 (9.5)	144 (25.7)	169 (13.9)	73 (8.5)	203 (17.4)	< 0.0001
Tacrolimus	212 (4.5)	36 (3.9)	15 (2.7)	49 (4.0)	33 (3.9)	79 (6.8)	0.0003
Glucocorticoid use, *n* (%)^a^	4143 (87.5)	817 (87.5)	486 (86.6)	1081 (88.8)	672 (78.3)	1087 (93.3)	< 0.0001
LTBI prophylaxis, *n* (%)	1168 (24.7)	279 (29.9)	113 (20.1)	298 (24.5)	182 (21.2)	296 (25.4)	< 0.0001
Entry year, *n* (%)							< 0.0001
2013	85 (1.8)	16 (1.7)	18 (3.2)	14 (1.2)	11 (1.3)	26 (2.2)	
2014	1208 (25.5)	321 (34.4)	187 (33.3)	271 (22.3)	182 (21.2)	247 (21.2)	
2015	956 (20.2)	181 (19.4)	127 (22.6)	225 (18.5)	179 (20.9)	244 (20.9)	
2016	1002 (21.2)	164 (17.6)	100 (17.8)	291 (23.9)	174 (20.3)	273 (23.4)	
2017	1033 (21.8)	163 (17.5)	89 (15.9)	276 (22.7)	222 (25.9)	283 (24.3)	
2018	452 (9.5)	89 (9.5)	40 (7.1)	141 (11.6)	90 (10.5)	92 (7.9)	

*TNF* tumor necrosis factor, *ETA* etanercept, *INF* infliximab, *ADA* adalimumab, *GOL* golimumab, *TOC* tocilizumab, *SD* standard deviation, *DMARDs* disease-modifying anti-rheumatic drugs, *LTBI* latent tuberculosis infection

^a^The use of conventional DMARDs and glucocorticoid was evaluated during 3 months after initiating biologic drugs

### Incidence of TB after biologic therapy

**Fig. 2:**
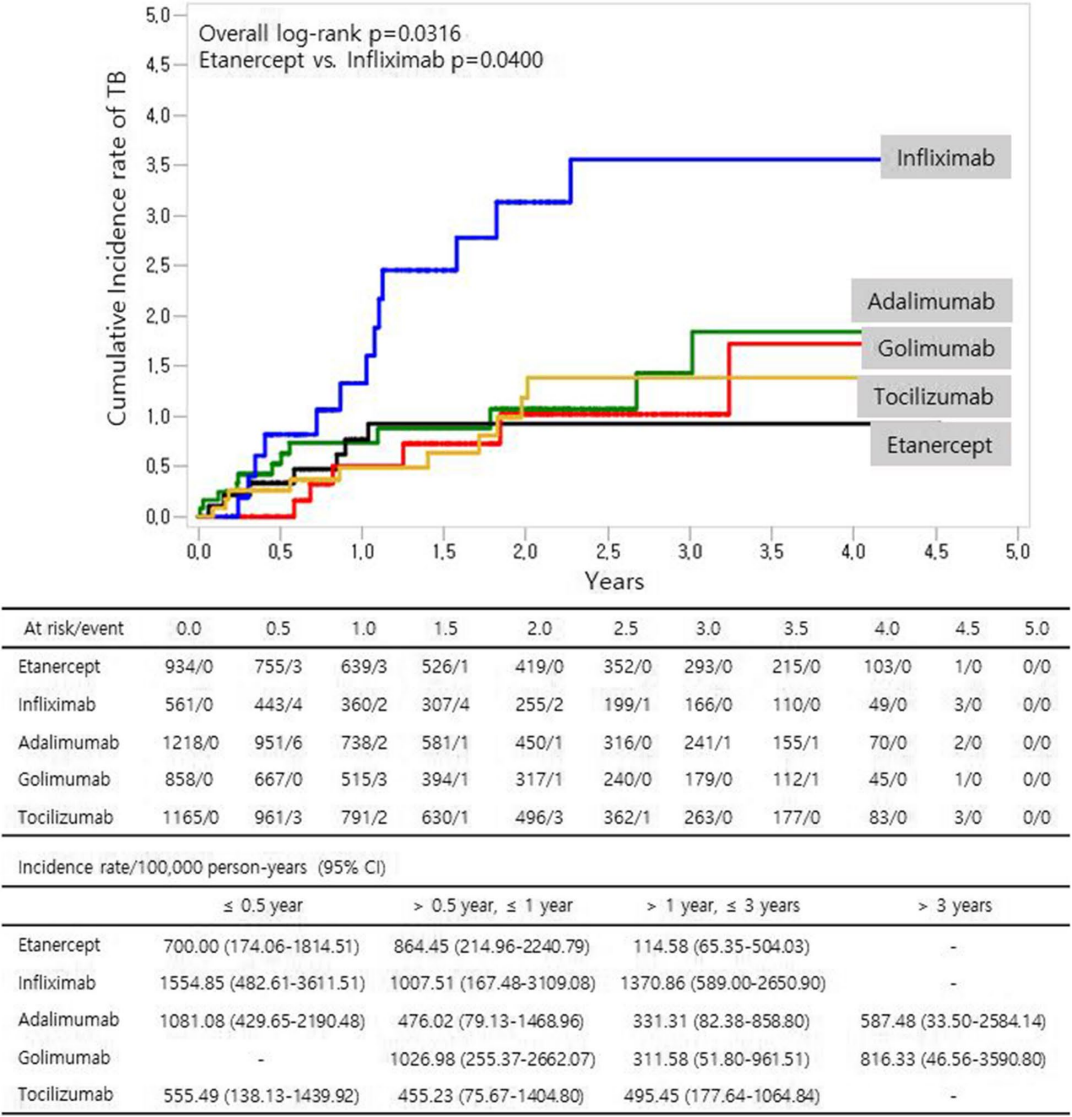
Cumulative incidence of tuberculosis (TB) in patients with rheumatoid arthritis (RA) who received biologic therapy. The cumulative incidence rate of TB was evaluated using the Kaplan–Meier method, and the differences among biologic therapies were compared using the log-rank test with multiple comparison adjustment. Infliximab showed a significantly higher risk of TB than etanercept (*P* = 0.04). The table shows the incidence rate of TB in patients with RA stratified by follow-up duration (< 0.5, 0.5–1, 1–3, and ≥ 3 years) CI, confidence interval

The median follow-up duration was 569 days (interquartile range 230–1075 days) in the whole study population. The total follow-up time was 8650.8 person-years, and the incidence rate of TB was 554.9 per 100,000 person-years. The crude incidence rate of TB was the highest in patients treated with infliximab, followed by those treated with adalimumab, tocilizumab, golimumab, and etanercept. The cumulative incidence rate of TB was also significantly higher in patients treated with infliximab than in those treated with etanercept (*P* = 0.04, Fig. 2). After adjusting for age, sex, and entry year, the IRR was significantly higher in the infliximab group (IRR 3.06 [95% CI 1.22–7.69]) than in the etanercept group (Table 2). The incidence of TB in all patients including switchers showed a similar pattern for each biologic agent (Supplementary Table 2). In all patients including switchers, we further analyzed the risk of TB according to the previous use of other biologic therapy within 6 months. The previous use of other biologic therapy showed no significant impact on developing TB in both TNF inhibitor users and tocilizumab users (Supplementary Table 3).

**Table 2:** Risk of tuberculosis in patients with rheumatoid arthritis treated with biologic therapy

			Anti-TNF			
	All (*n* = 4736)	ETA (*n* = 934)	INF (*n* = 561)	ADA (*n* = 1,218)	GOL (*n* = 858)	TOC (*n* = 1165)
Duration of follow-up (days), median (IQR)	569 (230–1075)	640 (256–1225)	642 (222–1174)	510 (216–931)	475 (204–984)	612 (281–1061)
Person-years	8650.8	1879.7	1087.1	2050.9	1447.5	2185.6
Case of TB, *n*	48	7	13	12	6	10
Rate/100,000 person-years (95% CI)	554.9 (412.3–727.0)	372.4 (160.0–720.1)	1195.8 (657.8–1968.5)	585.1 (313.3–981.2)	414.5 (164.7–839.9)	457.5 (229.3–802.4)
Adjusted IRR^a^		1.00 (ref)	3.06 (1.22–7.69)	1.69 (0.66–4.33)	1.22 (0.41–3.67)	1.25 (0.47–3.31)

*TNF* tumor necrosis factor, *ETA* etanercept, *INF* infliximab, *ADA* adalimumab, *GOL* golimumab, *TOC* tocilizumab, *IQR* interquartile range, *TB* tuberculosis, *CI* confidence interval, *IRR* incidence rate ratio

^a^The IRR was adjusted for age, sex, and entry year

### Difference in TB incidence according to LTBI treatment

The risk of TB was separately evaluated according to whether patients were treated for LTBI or not. Because interferon-gamma releasing assay and chest radiography are mandatory before biologic therapy in South Korea, the screening method for LTBI was considered to be appropriate. According to the local guideline [22], TB prophylaxis was given to all patients with evidence of LTBI. Of the total of 4763 patients, 1168 were treated for LTBI and 3568 did not receive TB prophylaxis because of a lack of evidence of LTBI. The median follow-up duration was 553 days in patients without evidence of LTBI and 612 days in patients treated for LTBI.

New case of TB was detected in 34 patients without evidence of LTBI. The crude incidence rate of TB was the lowest in patients treated with etanercept and tocilizumab, followed by those treated with golimumab, adalimumab, and infliximab (Table 3). The adjusted IRRs showed a similar pattern with the IRRs obtained from the whole study population. Infliximab was associated with a higher risk of TB compared to etanercept (3.65 [95% CI 1.14–11.7]). The difference between etanercept and infliximab was also detected in the analysis including switchers (Supplementary Table 4).

**Table 3:** Risk of tuberculosis in patients with rheumatoid arthritis according to treatment for latent tuberculosis infection

			Anti-TNF			
	All	ETA	INF	ADA	GOL	TOC
**In patients without evidence of latent tuberculosis infection**						
	(*n* = 3568)	(*n* = 655)	(*n* = 448)	(*n* = 920)	(*n* = 676)	(*n* = 869)
Duration of follow-up (days), median (IQR)	553 (227–1050)	629 (253–1196)	583 (216–1161)	510 (216–925)	470 (203–958)	608 (271–1061)
Person-years	6421.3	1295.9	844.4	1549.0	1113.1	1618.9
Case of TB, *n*	34	4	10	9	6	5
Rate/100,000 person-years (95% CI)	529.5 (370.9–727.9)	308.7 (95.8–717.0)	1184.2 (593.4–2077.9)	581.0 (279.2–1047.3)	539.0 (214.2–1092.2)	308.9 (110.7–663.8)
Adjusted IRR^a^		1.00 (ref)	3.65 (1.14–11.7)	2.18 (0.66–7.14)	2.00 (0.56–7.16)	1.07 (0.29–4.04)
**In patients who were treated for latent tuberculosis infection before biologic therapy**						
	(*n* = 1168)	(*n* = 279)	(*n* = 113)	(*n* = 298)	(*n* = 182)	(*n* = 296)
Duration of follow-up (days), median (IQR)	612 (248–1134)	680 (257–1315)	803 (271–1230)	506 (221–963)	549 (213–1144)	668 (304–1066)
Person-years	2229.5	583.8	242.7	501.9	334.3	566.8
Case of TB, *n*	14	3	3	3	0	5
Rate/100,000 person-years (95% CI)	627.9 (353.7–1016.6)	513.9 (127.8–1332.0)	1236.2 (307.4–3204.4)	597.7 (148.6–1549.4)	_	882.2 (316.3–1896.0)
Adjusted IRR		1.00 (ref)	2.54 (0.50–12.96)	0.93 (0.18–4.70)	_	1.35 (0.32–5.80)

*TNF* tumor necrosis factor, *ETA* etanercept, *INF* infliximab, *ADA* adalimumab, *GOL* golimumab, *TOC* tocilizumab, *IQR* interquartile range, *TB* tuberculosis, *CI* confidence interval, *IRR* incidence rate ratio

^a^The IRR was adjusted for age, sex, and entry year

Among patients who received TB prophylaxis before biologic therapy, 14 patients received anti-TB treatment after initiating biologic drugs. The incidence rate of TB was higher in patients with LTBI than in those without evidence of LTBI (627.9 vs. 529.5 per 100,000 person-years). The TB incidence was also the highest in the infliximab group and the lowest in the etanercept group (Table 3). However, the adjusted IRRs showed no significant difference among all TNF inhibitors and tocilizumab in patients treated for LTBI. Tocilizumab showed a higher incidence of TB in patients treated for LTBI than in those without LTBI treatment, although with no statistical significance (adjusted IRR 2.42 [95% CI 0.66–8.88]). Other biologic agents showed a similar incidence of TB between patients with and those without LTBI treatment.

## Discussion

The differential risk of TB among TNF inhibitors has been intensively studied in patients with RA. The present real-world nationwide data also showed that the risk of TB was the highest in the infliximab group and the lowest in the etanercept group. Tocilizumab, which has a different mode of action than TNF blockade, had a similar risk of TB to that of etanercept. The drug-specific risk of biologic therapy on TB development became inapparent in patients who received LTBI treatment.

Given that the annual incidence of TB was approximately 70 per 100,000 persons in the general population of South Korea [20], patients treated with biologic therapy showed a significantly high risk of TB. However, the TB incidence after biologic therapy was markedly reduced compared with that reported in a previous nationwide study (1143 per 100,000 person-years in patients with RA treated with TNF inhibitors) [15]. The main cause of the drastic decrease is probably the mandatory evaluation and treatment for LTBI. In the previous study conducted from 2005 to 2009, patients treated for LTBI accounted for 20.5% of all patients who used TNF inhibitors and only 14.1% of patients with RA were treated for LTBI [15]. However, the present study included 1168 (24.7%) patients with RA who received treatment for LTBI. The prevalence of LTBI is similar to that estimated by interferon-gamma releasing assay in the general population at a high risk for TB, which was 25.4% among health-care and nursery workers in their 50 s [19].

Unlike in earlier studies [13, 14], the drug-associated risk of TB was not significantly different between etanercept and adalimumab/golimumab in this study. This finding may be associated with the high prevalence of LTBI in patients treated with etanercept. Patients diagnosed with LTBI were more likely to be treated with etanercept owing to concerns about reactivation of LTBI. Real-world data in Taiwan and Hong Kong, which are countries with intermediate TB burden, also showed a similar incidence of TB in patients with RA who used etanercept and adalimumab [23, 24]. Golimumab, the latest TNF inhibitor, showed a comparable risk of TB to that of etanercept regardless of LTBI treatment. Although golimumab is expected to have a similar safety profile to adalimumab, the association of golimumab and TB has not been intensively investigated. To our knowledge, this is the first study to compare the risk of TB between golimumab and other TNF inhibitors in a nationwide sample. The reason for the differential risk among monoclonal anti-TNF antibodies is unclear. Drug-specific characteristics, such as administration route, half-life, and molecular features, might affect the differential risk of TB. To explain the differential risk of TB among monoclonal anti-TNF antibodies, further studies are warranted.

This nationwide study also compared the risk of TB between TNF inhibitors and tocilizumab. Tocilizumab is a monoclonal antibody targeted to the IL-6 receptor to block the inflammatory activity of IL-6. Despite the wide use of tocilizumab in patients with RA, the risk of TB in patients treated with tocilizumab was largely unknown. Although a nationwide study was performed to compare the risk of TB between tocilizumab and TNF inhibitors in Taiwan with an intermediate TB burden, TB was not detected in tocilizumab users [23]. In post-marketing surveillance in Japan, the TB incidence in patients treated with tocilizumab was similar to that in patients treated with TNF inhibitors [25]. However, a direct comparison between TNF inhibitors and tocilizumab has not been performed, and the risk of TB associated with tocilizumab was evaluated only in Japan with low TB incidence.

The present study provides clinical insight on the association between IL-6 and TB. The increased risk of TB following anti-TNF therapy was also observed in tocilizumab users. Although the role of IL-6 in the immunity against TB has not been established, the association between IL-6 polymorphism and TB susceptibility suggests that IL-6 have a role in the pathogenesis of TB [26]. In previous experimental study, the absence of IL-6 was associated with delayed interferon-gamma production and early increase in bacterial load [27]. Interestingly, the incidence of TB after tocilizumab treatment was higher in patients treated for LTBI than that in those without evidence of LTBI. This finding suggests that the effect of IL-6 blockade may be different between de novo infection of TB and reactivation of chronic latent TB.

The incidence of TB was 529.5 and 627.9 per 100,000 person-years in patients without and with LTBI treatment, respectively. The risk of TB was not significantly different between patients treated for LTBI and those without evidence of LTBI. Recently, Lee et al. reported the incidence of TB after anti-TNF therapy in South Korea as 1046/100,000 person-years in patients not treated for LTBI and 407/100,000 person-years in those treated for LTBI [28]. The discrepancy in the TB incidence may be due to differences in the study population and study period. The previous study included heterogeneous patients who received anti-TNF treatment, such as patients with RA, ankylosing spondylitis, psoriatic arthritis, and inflammatory bowel disease. Moreover, the study was conducted between 2011 and 2013, which was earlier than the current study. As the appropriate screening of LTBI became available and the rate of LTBI treatment increased over time, the gap in TB incidence according to LTBI treatment was markedly reduced.

In order to evaluate the drug-specific risk of TB while excluding the effects of previous biologic therapy, this study was performed in patients treated only with conventional DMARDs for at least 6 months prior to enrollment. However, switching biologic therapy is very common in patients with RA, especially longstanding RA. Given that each patient’s situation is diverse in real-world clinical practice, meticulous attention is required to assess the risk of TB following biologic therapy.

This study has several limitations. First, it was not feasible to adjust all covariates to conclude the effects of biologic therapy on TB risk. The clinical factors affecting TB development, such as nutritional status, socioeconomic status, comorbidities, and RA-specific features, were not fully evaluated owing to the limitation of the database. Additionally, the low incidence of TB limits further adjustment of other risk factors, such as concomitant medications. Second, the diagnosis of TB was determined according to the prescription of anti-TB drugs rather than through bacteriologic or pathologic confirmation. However, biologic therapy in patients with RA is usually performed in secondary or tertiary care institutes, and patients are sequentially followed up. Thus, misdiagnosis of TB or failure to detect TB is very rare in these patients. Third, the risk of TB was not evaluated in patients treated with other non-TNF biologic agents, including abatacept, rituximab, and Janus kinase inhibitors. Because the amount of other non-TNF biologic therapy was much smaller than that of TNF inhibitors and tocilizumab in South Korea, it is likely to yield unclear results. Further studies would be required to compare the safety of various biologic drugs if data are further accumulated. Finally, despite these limitations, this is the first study comparing the risk of TB in patients treated with TNF inhibitors and tocilizumab. Further, the incidence of TB was separately evaluated in patients with and without LTBI treatment.

## Conclusions

In conclusion, tocilizumab showed a comparable risk of TB to that of etanercept. Despite a different mechanism of action from TNF inhibitors, tocilizumab may confer a risk of developing TB in patients with RA. Infliximab may increase the risk of TB than other TNF inhibitors, especially in patients who were not treated for LTBI. Treatment of LTBI might obliterate the differential effect of biologic drugs on developing TB. Physicians should be aware of the importance of selecting a biologic agent and treating LTBI for the prevention of TB in all patients receiving biologic therapy.

## Supplementary Information

**Additional file 1: Figure S1.**

**Additional file 2:** **Table S1.** Clinical characteristics of patients with rheumatoid arthritis in a nationwide database (including switchers). **Table S2.** Risk of tuberculosis in patients with rheumatoid arthritis treated with biologic therapy (including switchers). **Table S3-1.** Risk of tuberculosis according to the use of other biologic therapy within the preceding 6 months. **Table S3-2.** Risk of tuberculosis according to the use of other biologic therapy within the preceding 6 months. **Table S4.** Risk of tuberculosis in patients with rheumatoid arthritis according to treatment of latent tuberculosis infection (including switchers).

## Data Availability

The data that support the findings of this study are available from the HIRA database but restrictions apply to the availability of these data, which were used under license for the current study, and so are not publicly available. Data are however available from the authors upon reasonable request and with permission of HIRA.

## Abbreviations

TNF: Tumor necrosis factor
TB: Tuberculosis
RA: Rheumatoid arthritis
LTBI: Latent tuberculosis infection
DMARD: Disease-modifying anti-rheumatic drug
HIRA: The Health Insurance Review and Assessment service
IRR: Incidence rate ratio
CI: Confidence interval
