# Supplementary figures and images for: Comparison of developing tuberculosis following tumor necrosis factor inhibition and interleukin-6 inhibition in patients with rheumatoid arthritis: a nationwide observational study in South Korea, 2013–2018
Source: Arthritis Res Ther. 2022 Jun 27;24:157. doi: 10.1186/s13075-022-02842-6 (PMC9235163; doi:10.1186/s13075-022-02842-6)

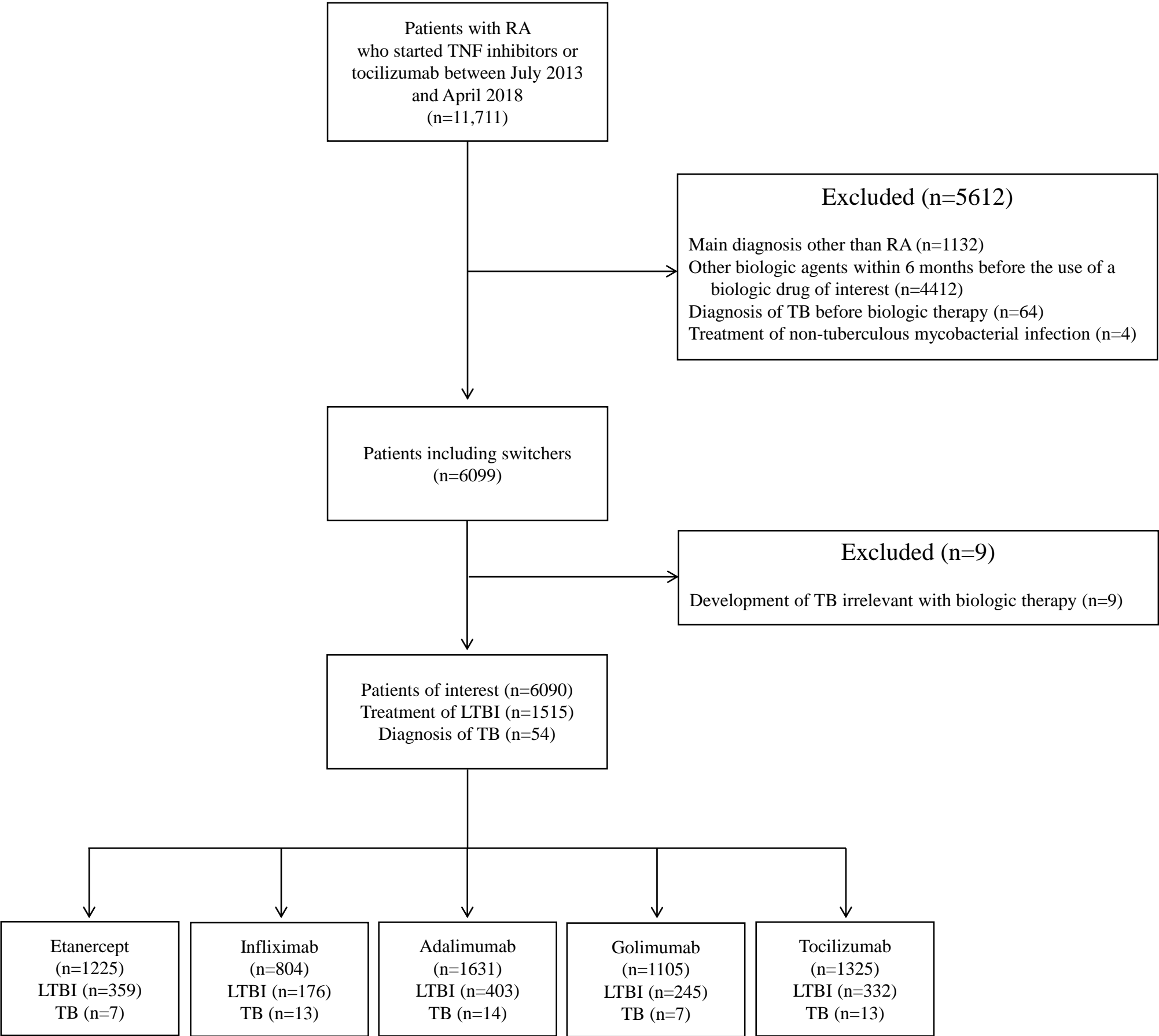

Supplement: Supplementary file 1 — Additional file 1: Figure S1. [file 13075_2022_2842_MOESM1_ESM.pdf]
