# Supplementary material for: Comparison of developing tuberculosis following tumor necrosis factor inhibition and interleukin-6 inhibition in patients with rheumatoid arthritis: a nationwide observational study in South Korea, 2013–2018
Source: Arthritis Res Ther. 2022 Jun 27;24:157. doi: 10.1186/s13075-022-02842-6 (PMC9235163; doi:10.1186/s13075-022-02842-6)
Supplement: Supplementary file 2 — Additional file 2: Table S1. Clinical characteristics of patients with rheumatoid arthritis in a nationwide database (including switchers). Table S2. Risk of tuberculosis in patients with rheumatoid arthritis treated with biologic therapy (including switchers). Table S3-1. Risk of tuberculosis according to the use of other biologic therapy within the preceding 6 months. Table S3-2. Risk of tuberculosis according to the use of other biologic therapy within the preceding 6 months. Table S4. Risk of tuberculosis in patients with rheumatoid arthritis according to treatment of latent tuberculosis infection (including switchers). [file 13075_2022_2842_MOESM2_ESM.docx]

**Supplementary Table 1.** Clinical characteristics of patients with rheumatoid arthritis in a nationwide database (including switchers)

|  |  | | Anti-TNF | | |  | | |  | |
| --- | --- | --- | --- | --- | --- | --- | --- | --- | --- | --- |
|  | All  (n=6090) | ETA  (n=1225) | | INF  (n=804) | ADA  (n=1631) | | GOL  (n=1105) | TOC  (n=1325) | | *P* value |
| Age (years), mean ± SD | 53.7 ± 13.3 | 53.6 ± 14.2 | | 54.7 ± 12.8 | 51.8 ± 13.6 | | 54.3 ± 13.0 | 55.1 ± 12.5 | | <0.0001 |
| Women, n (%) | 4926 (80.9) | 971 (79.3) | | 661 (82.2) | 1238 (78.7) | | 915 (82.8) | 1096 (82.7) | | 0.0083 |
| Combination of DMARDs, n (%)^a^  Methotrexate  Leflunomide  Tacrolimus | 5,103 (83.8)  875 (14.4)  317 (5.2) | 998 (81.5)  134 (10.9)  62 (5.1) | | 705 (87.7)  190 (23.6)  29 (3.6) | 1402 (86.0)  226 (13.9)  79 (4.8) | | 1002 (90.7)  93 (8.4)  52 (4.7) | 996 (75.2)  232 (17.5)  95 (7.2) | | <0.0001  <0.0001  0.0037 |
| Glucocorticoid use, n (%)^a^ | 5401 (88.7) | 1096 (89.5) | | 702 (87.3) | 1464 (89.8) | | 901 (81.5) | 1238 (93.4) | | <0.0001 |
| LTBI prophylaxis, n (%) | 1515 (24.9) | 359 (29.3) | | 176 (21.9) | 403 (24.7) | | 245 (22.2) | 332 (25.1) | | <0.0001 |
| Entry year, n (%)  2013  2014  2015  2016  2017  2018 | 127 (2.1)  1729 (28.4)  1295 (21.3)  1281 (21.0)  1195 (19.6)  463 (7.6) | 27 (2.2)  437 (35.7)  262 (21.4)  208 (17.0)  200 (16.3)  91 (7.4) | | 26 (3.2)  312 (38.8)  189 (23.5)  137 (17.0)  100 (12.4)  40 (5.0) | 29 (1.8)  418 (25.6)  307 (18.8)  390 (23.9)  340 (20.9)  147 (9.0) | | 15 (1.4)  258 (23.4)  253 (22.9)  236 (21.4)  251 (22.7)  92 (8.3) | 30 (2.3)  304 (22.9)  284 (21.4)  310 (23.4)  304 (22.9)  93 (7.0) | | <0.0001 |

TNF, tumor necrosis factor; ETA, etanercept; INF, infliximab; ADA, adalimumab; GOL, golimumab; TOC, tocilizumab; SD, standard deviation; DMARDs, disease modifying anti-rheumatic drugs; LTBI, latent tuberculosis infection.

^a^ The use of conventional DMARDs and glucocorticoid was evaluated during 3 months after initiating biologic drugs.

**Supplementary 2.** Risk of tuberculosis in patients with rheumatoid arthritis treated with biologic therapy (including switchers)

|  |  | | Anti-TNF | |  | |
| --- | --- | --- | --- | --- | --- | --- |
|  | All  (n=6090) | ETA  (n=1225) | INF  (n=804) | ADA  (n=1631) | GOL  (n=1105) | TOC  (n=1325) |
| Duration of follow-up (days), median (IQR) | 677 (294 – 1157) | 736  (349 – 1269) | 837  (329 – 1304) | 615  (261 – 1065) | 610  (258 – 1068) | 657  (314 – 1084) |
| Person-years | 12216.3 | 2656.5 | 1814.3 | 3091.2 | 2072.4 | 2581.9 |
| Case of TB, n | 54 | 7 | 13 | 14 | 7 | 13 |
| Rate/100,000 person-years (95% CI) | 442.0  (334.4 – 570.6) | 263.5  (113.2 – 509.6) | 716.5  (394.2 – 1179.5) | 452.9  (255.1 – 733.2) | 337.8  (145.1 – 653.2) | 503.5  (277.0 – 828.8) |
| Adjusted IRR^a^ |  | 1.00 (ref) | 2.76  (1.10 – 6.93) | 1.90  (0.76 – 4.74) | 1.38  (0.48 – 3.94) | 1.97  (0.78 – 4.96) |

TNF, tumor necrosis factor; ETA, etanercept; INF, infliximab; ADA, adalimumab; GOL, golimumab; TOC, tocilizumab; IQR, interquartile range; TB, tuberculosis; CI, confidence interval; IRR, incidence rate ratio.

^a^ The IRR was adjusted for age, sex, and entry year.

**Supplementary Table 3-1.** Risk of tuberculosis according to the use of other biologic therapy within the preceding 6 months

|  | All  (n=5589) | Without previous biologic therapy  (n=4736) | With previous biologic therapy  (n=853) |
| --- | --- | --- | --- |
| Duration of follow-up (days), median (IQR) | 573 (232 – 1057) | 569 (230 – 1075) | 583 (243 – 1009) |
| Person-years | 10164.4 | 8650.8 | 1513.6 |
| Case of TB, n | 54 | 48 | 6 |
| Rate/100,000 person-years (95% CI) | 531.3 (401.9 – 685.8) | 554.9 (412.3 – 727.0) | 396.4 (157.5 – 803.2) |
| Adjusted IRR^a^ |  | 1.00 (ref) | 0.76 (0.32 – 1.80) |

IQR, interquartile range; TB, tuberculosis; CI, confidence interval; IRR, incidence rate ratio.

^a^ The IRR was adjusted for age, sex, and entry year.

**Supplementary Table 3-2.** Risk of tuberculosis according to the use of other biologic therapy within the preceding 6 months

|  | Anti-TNF  (n=4010) | | TOC  (n=1579) | |
| --- | --- | --- | --- | --- |
|  | Without previous biologic therapy  (n=3571) | With previous biologic therapy  (n=439) | Without previous biologic therapy  (n=1165) | With previous biologic therapy  (n=414) |
| Duration of follow-up (days), median (IQR) | 557 (219 – 1084) | 517 (229 – 1026) | 612 (281 – 1061) | 667 (265 – 1000) |
| Person-years | 6465.2 | 757.2 | 2185.6 | 756.4 |
| Case of TB, n | 38 | 3 | 10 | 3 |
| Rate/100,000 person-years (95% CI) | 587.8 (420.1 – 794.9) | 396.2 (98.5 – 1027.0) | 457.5 (229.3 – 802.4) | 396.6 (98.6 – 1028.1) |
| Adjusted IRR^a^ | 1.00 (ref) | 0.80 (0.24 – 2.61) | 0.76 (0.38 – 1.53) | 0.66 (0.20 – 2.14) |

TNF, tumor necrosis factor; TOC, tocilizumab; IQR, interquartile range; TB, tuberculosis; CI, confidence interval; IRR, incidence rate ratio.

^a^ The IRR was adjusted for age, sex, and entry year.

**Supplementary Table 4.** Risk of tuberculosis in patients with rheumatoid arthritis according to treatment of latent tuberculosis infection (including switchers)

|  |  | | Anti-TNF | |  | |
| --- | --- | --- | --- | --- | --- | --- |
|  | All | ETA | INF | ADA | GOL | TOC |
| **In patients without evidence of latent tuberculosis infection** | | | | | | |
|  | (n=4575) | (n=866) | (n=628) | (n=1228) | (n=860) | (n=993) |
| Duration of follow-up (days), median (IQR) | 663  (286 – 1146) | 699  (350 – 1228) | 803  (301 – 1278) | 620  (260 – 1075) | 575  (244 – 1043) | 650  (313 – 1085) |
| Person-years | 9057.9 | 1843.9 | 1375.5 | 2332.7 | 1576.5 | 1929.3 |
| Case of TB, n | 34 | 4 | 10 | 9 | 6 | 5 |
| Rate/100,000 person-years (95% CI) | 419.5  (299.9 – 567.4) | 216.9  (67.3 – 503.9) | 727.0  (364.3 – 1275.0) | 471.6  (244.8 – 807.5) | 444.0  (190.8 – 858.6) | 311.0  (123.6 – 630.1) |
| Adjusted IRR^1^ |  | 1.00 (ref) | 3.43  (1.07 – 10.95) | 2.58  (0.82 – 8.17) | 2.26  (0.66 – 7.74) | 1.55  (0.43 – 5.52) |
| **In patients who were treated for latent tuberculosis infection before biologic therapy** | | | | | | |
|  | (n=1515) | (n=359) | (n=176) | (n=403) | (n=245) | (n=332) |
| Duration of follow-up (days), median (IQR) | 716  (314 – 1212) | 821  (326 – 1342) | 967  (436 – 1374) | 593  (275 – 1043) | 685  (300 – 1157) | 674  (327 – 1082) |
| Person-years | 3158.5 | 812.6 | 438.8 | 725.5 | 495.9 | 652.6 |
| Case of TB, n | 16 | 3 | 3 | 3 | 0 | 7 |
| Rate/100,000 person-years (95% CI) | 506.6  (297.1 – 796.8) | 369.2  (91.8 – 956.9) | 683.6  (170.0 – 1772.1) | 395.5  (98.4 – 1025.3) | _ | 1072.6  (460.9 – 2074.2) |
| Adjusted IRR^1^ |  | 1.00 (ref) | 2.12  (0.42 – 10.56) | 0.92  (0.18 – 4.64) | _ | 2.36  (0.60 – 9.30) |

TNF, tumor necrosis factor; ETA, etanercept; INF, infliximab; ADA, adalimumab; GOL, golimumab; TOC, tocilizumab; IQR, interquartile range; TB, tuberculosis; CI, confidence interval; IRR, incidence rate ratio.

^1^ The IRR was adjusted for age, sex, and entry year.
